# Nucleosome deposition and DNA methylation at coding region boundaries

**DOI:** 10.1186/gb-2009-10-9-r89

**Published:** 2009-09-01

**Authors:** Jung Kyoon Choi, Jae-Bum Bae, Jaemyun Lyu, Tae-Yoon Kim, Young-Joon Kim

**Affiliations:** 1 Department of Biochemistry, College of Life Science and Technology, Yonsei University, 134 Sinchon-dong, Seodaemun-gu, Seoul, Korea; 2 Laboratory of Dermato-Immunology, The Catholic University of Korea, 505 Banpo-dong, Seocho-gu, Seoul, Korea

## Abstract

**Background:**

Nucleosome deposition downstream of transcription initiation and DNA methylation in the gene body suggest that control of transcription elongation is a key aspect of epigenetic regulation.

**Results:**

Here we report a genome-wide observation of distinct peaks of nucleosomes and methylation at both ends of a protein coding unit. Elongating polymerases tend to pause near both coding ends immediately upstream of the epigenetic peaks, causing a significant reduction in elongation efficiency. Conserved features in underlying protein coding sequences seem to dictate their evolutionary conservation across multiple species. The nucleosomal and methylation marks are commonly associated with high sequence-encoded DNA-bending propensity but differentially with CpG density. As the gene grows longer, the epigenetic codes seem to be shifted from variable inner sequences toward boundary regions, rendering the peaks more prominent in higher organisms.

**Conclusions:**

Recent studies suggest that epigenetic inhibition of transcription elongation facilitates the inclusion of constitutive exons during RNA splicing. The epigenetic marks we identified here seem to secure the first and last coding exons from exon skipping as they are indispensable for accurate translation.

## Abstract 2

Nucleosomes and methylation have been observed to peak at both ends of protein coding units in a genome-wide survey.

## Background

Recent epigenomic studies point out that epigenetic control of transcription elongation is a widespread regulatory mechanism. Intragenic DNA methylation occurs at higher density [1-3] and has a larger effect on expression level than promoter methylation [4], inhibiting transcription elongation in filamentous fungi [5,6], plant protoplasts [7], *Arabidopsis *[2], and mammalian cells [8]. Remarkably, methylation of protein coding regions alone can inhibit gene expression and its inhibitory effects are larger when it occurs nearer the start codon [7]. Intriguingly, the methylation map of rice chromosomes exhibits single peaks near start codons [4]. It is probable that the methylation peak near the start codon exerts major inhibitory effects on transcription elongation.

It may be that specific organization of nucleosomes surrounding the coding start region is also important in regulating transcription elongation. The maps of H2A.Z-containing nucleosomes in yeast and fly reveal conserved positioning of a nucleosome downstream of the transcription start site (TSS; the +1 nucleosome) [9,10]. Notably, another yeast study provides a hint that the +1 nucleosome peaks very close to the start codon [11]. In addition, there seems to be a nucleosomal peak prior to the 3' end of the yeast open reading frame (ORF) as well [12]. The +1 nucleosome of fly is located further from the TSS than that of yeast [10]. However, fly nucleosome patterns surrounding the start and stop codons have never been examined.

Taken together, anecdotal findings in different species suggest a role for coding region boundaries in maintaining nucleosome deposition and DNA methylation. From a mechanistic perspective, the epigenetic marks can act as 'roadblocks' to RNA polymerase II (Pol II) progression. However, previous studies have focused on transcription initiation or termination, but not elongation. In this study, we attempt to provide insight into elongation inhibition surrounding translation start and end sites, which has never been observed. Given the impact of DNA methylation on nucleosome formation [13], correlations or differences between the two epigenetic marks also deserve systematic investigation.

## Results

We first surveyed published nucleosome positions in yeast and fly [9,10]. When aligned at the TSS, there is a significant difference in +1 nucleosome position between the two species [10] (Additional data file 1a). However, aligning by the coding region places the first coding nucleosome in similar positions in the two species - that is, just downstream of the start codon (Additional data file 1b). We also identified a highly conserved nucleosome immediately upstream of the 3' coding end in both species (Additional data file 1c).

**Figure 1:**
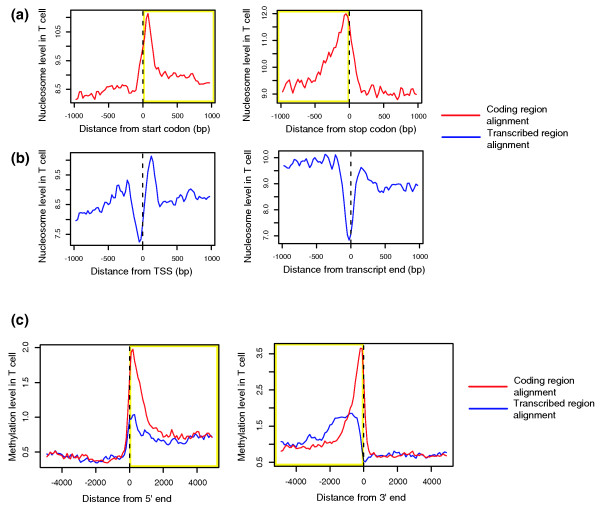
Epigenetic peaks near coding region boundaries. **(a, b) **Genome-wide average of nucleosome occupancy in T cells for genes aligned at the (a) coding ends or (b) transcript ends. The inner coding region is outlined in yellow. **(c) **Genome-wide average of methylation level in T cells for genes aligned at the coding ends or transcript ends. The inner coding and transcript regions are outlined in yellow.

Analyzing the H2A.Z map for human T cells [14] also revealed nucleosomal peaks just downstream of start codons and just upstream of stop codons, marking both ends of the coding sequences (Figure 1a). Meanwhile, boundaries at both ends of transcripts are tightly coupled with nucleosome-free regions, potentially allowing access of the initiation and termination complex (Figure 1b). The nucleosome-free region at the TSS was followed by the +1 nucleosome. However, the association of the +1 nucleosome and the TSS appears to be weaker than that of the first coding nucleosome and the start codon. The patterns of nucleosome positioning for some individual genes are shown in Additional data file 2.

We carried out Solexa sequencing of methylated DNA from human T cells and found methylation peaks at the exact same positions (Figure 1c). We also profiled the mouse liver and found the same patterns (Additional data file 3). Together, the nucleosomal peaks are observed in human, fly, and yeast, and the methylation peaks in human, mouse, and plants.

**Figure 2:**
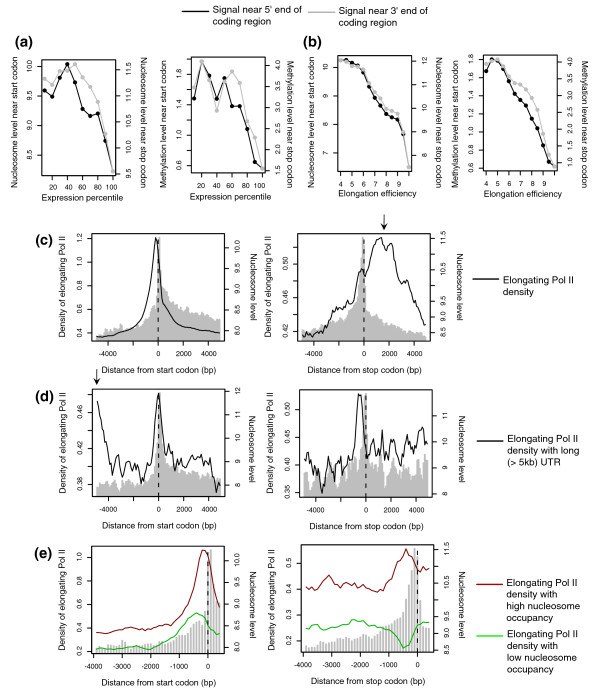
Correlation of elongation inhibition with epigenetic peaks. **(a, b) **The average of nucleosome level (left panel) and methylation level (right panel) were plotted (a) within each expression percentile and (b) within each bin of elongation efficiency. The epigenetic marks near the start codon are scaled on the left side (black curve) and those near the stop codon on the right side (gray curve). **(c-e) **Density of Ser5-phosphorylated Pol II (black trace) and nucleosome level (gray) surrounding the start codon (left panel) or stop codon (right panel) for (c) all genes, (d) genes with a long (> 5 kb) 5' UTR (left panel) or 3' UTR (right panel), and (e) genes with high- or low-occupancy boundary nucleosomes (top 10% or bottom 10%). The Pol II scale is on the left side and the nucleosome scale is on the right side.

To examine their role in regulating transcription, we first related the level of the epigenetic peaks to expression level. We found that highly expressed genes are depleted of the epigenetic peaks (Figure 2a), consistent with findings of a nucleosomal barrier against high transcription rate [15]. However, the overall correlation was not strong. We then estimated elongation efficiency as mRNA production per unit density of elongating Pol II. Upon initiation, Pol II is phosphorylated at Ser5 in its carboxy-terminal domain, switching to an elongation-competent form. Thus, we calculated the ratio of expression level to the density of Ser5-phosphorylated Pol II within the transcript body. Genes with high elongation efficiency will show high expression levels even with a low density of elongating Pol II across the transcribed region, and the opposite for low elongation efficiency. A strong association was found between the level of the epigenetic peaks and elongation efficiency (Figure 2b; Additional data file 4).

Without any interference, elongating Pol II should be distributed evenly across the transcript body except at the initiating and terminating sites. Faced with roadblocks, however, Pol II pauses and a pileup of Pol II forms, which can be observed as a peak of Pol II density. Thus, to demonstrate Pol II pausing at epigenetic marks, three criteria should be met: the presence of a Pol II peak; the presence of an epigenetic peak; and a correspondence between the positions of the two peaks.

Elongating Pol II appears to pile up immediately upstream of the nucleosomal peaks at both ends of protein coding units (Figure 2c), satisfying the three criteria. However, there seem to be confounding effects from Pol II enriched at nearby transcription initiation and termination sites. For example, the Pol II tail downstream of the stop codon (arrow above the right panel of Figure 2c) might reflect Pol II awaiting to be released from the transcription termination site. We thus selected genes with a long (> 5 kb) 5' untranslated region (UTR) and examined Pol II density around the TSS and start codon separately. Both unphosphorylated and elongating Pol II were enriched at the TSS, but only elongating Pol II showed high downstream density (Additional data file 5) with a pileup upstream of the start codon (left panel of Figure 2d). Another Pol II peak upstream of the start codon (arrow above the left panel of Figure 2d) seems to reflect Pol II at the TSS. A pileup of Pol II was also found before a long (> 5 kb) 3' UTR (right panel of Figure 2d), indicative of Pol II blockage that occurs independently of the transcription termination site.

Pol II pausing was not observed with low nucleosome occupancy (Figure 2e), indicating that elongating Pol II is indeed impeded by the boundary nucleosome. To observe the specific effect of the boundary nucleosome, we computed relative occupancy at the coding end compared to the surrounding region. Higher or lower nucleosome occupancy near the coding end directly led to higher or lower Pol II density in the immediate upstream region (Additional data file 6).

We roughly estimated the percentage of genes that are affected by Pol II pausing by comparing the average Pol II density around boundaries and that across surrounding regions. We found that 54% of genes exhibit higher Pol II density near the start codon than in the flanking region and 41% of genes have a Pol II peak near the stop codon.

Nucleosome positioning is governed by DNA sequences [11,16]. Methylation level is dependent on the CpG content of the target sequence [17]. Given the distinctive patterns of nucleosome positioning and methylation maintained in specific regions among different species, there should be strong constraints on the underlying DNA sequences. Being under strong natural selection, protein coding sequences could be better candidates than UTRs for conserved epigenetic targets downstream of transcription initiation and upstream of termination. Coding region boundaries might be subject to considerable negative selection that purifies sequence changes that are detrimental to nucleosome deposition or DNA methylation.

**Figure 3:**
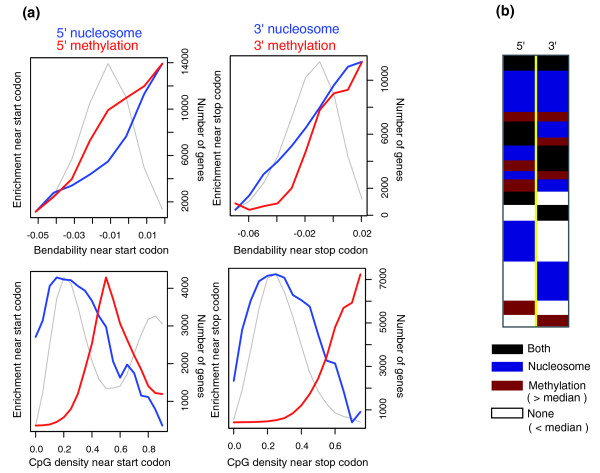
Correlation of genetic and epigenetic characteristics at coding region boundaries. **(a) **Bending propensity and CpG density were calculated for flanking sequences downstream of the start codon or upstream of the stop codon. The number of genes (gray curve measured on the right scale), methylation level (red curve), and nucleosome level (blue curve) were obtained according to the bendability (upper panels) and CpG density (lower panels) near the start codon (left panels) and stop codon (right panels). **(b) **A total of 25,883 genes were clustered by the strength of the marks near their coding ends. Marks with strengths higher than the median level of all genes were considered significant.

We examined two sequence characteristics deemed to be involved in epigenetic programming: DNA-bending propensity and CpG density. DNA-bending propensity, the ability of nucleotide sequences to wrap around a histone complex, is an important determinant of nucleosome formation [18,19]. DNase I digestion experiments indicate that bending parameters for the start codon and three stop codons are among the 8 highest out of those for the 32 trinucleotides [20]. Therefore, they can significantly contribute to the high bendability of coding boundaries (Additional data file 7). The boundary sequences with higher bendability tend to be more enriched for nucleosomes (upper panel in Figure 3a). Unexpectedly, DNA methylation level was also proportional to bending propensity.

CpG density should be a determinant of methylation level. The boundary sequences with intermediate CpG density were densely methylated (lower panel in Figure 3a). In contrast, nucleosome occupancy was dominant among genes with lower CpG density. While the two marks commonly have affinity for base compositions with high bending propensity, DNA methylation at CpG sites might affect structural DNA bending and nucleosome formation. The proportions of genes marked by both nucleosomes and methylation or by just one of these are shown in Figure 3b. More genes are specifically marked by nucleosomes than methylation, possibly because many boundary regions have relatively low CpG density (gray curve in lower panels of Figure 3a).

A group of genes had highest CpG density at the 5' end (gray curve at CpG density > 0.8 in bottom left panel of Figure 3a). These genes showed a markedly reduced level of DNA methylation, reflecting the fact that CpG islands are typically unmethylated. Indeed, 97.2% of these genes contained a CpG island within their promoter (-1,000 bp to 500 bp from the TSS) and 92.8% had a short (< 500 bp) UTR (*P *< 10^-100^), an indication that promoter CpG islands are overlapping or located very close to the start codon. These genes exhibited high expression compared to the rest of the genes (*P *< 10^-80^), even higher than the genes with a promoter CpG island (*P *= 1.4 × 10^-10^) (Additional data file 8), indicating additional effects of elongation control.

**Figure 4:**
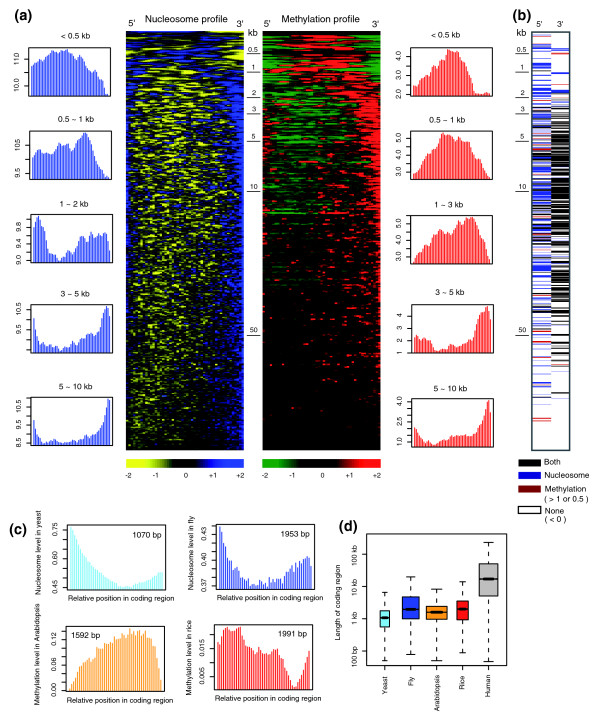
Epigenetic characteristics within coding regions of varying size. **(a) **Heatmaps showing the coding-region profiles of nucleosomes (left side) or methylation (right side) averaged over 100 neighboring genes ordered by size. A total of 25,883 genes were used. Each row represents a mean-centered profile for each gene. The average profile of genes of similar size is given on the left side (for nucleosome level) and the right side (for methylation level) of the heatmap. **(b) **A combined profile of the heatmap signatures of the two marks at each end (marks > 0.5 at the 5' end and > 1 at the 3' end were considered significant). **(c) **Genome-wide average of nucleosome occupancy in yeast and fly (upper panels) and methylation level in *Arabidopsis *and rice (lower panels) according to relative positions within the coding region. The median length of coding regions is shown. **(d) **Size of coding regions from each species. The box width is proportional to the square root of the number of measured genes.

Next, we explored the intragenic distribution of the marks. Although significantly higher than its flanking region, the 5' peak is generally lower than the 3' peak (Additional data file 9a). Meanwhile, k-means clustering shows that most genes have higher peaks at both ends compared to the central region (Additional data file 9b). We then examined these patterns according to the size of the coding region (Figure 4a). We found that genes with a short coding sequence (< 1 kb) have nucleosomes and methylation in their inner region over a large portion of the gene body. In particular, their 3' ends lack both marks, in sharp contrast to most other genes, in which the marks are shifted toward both ends with a bias toward the 3' end. Unlike at the 5' end, both marks commonly peaked at the 3' end, especially in many genes of intermediate size (Figure 4b).

Notably, nucleosome composition within the coding region of yeast or fly genes is not sharply shifted to the coding boundaries - the 3' peak is especially not very prominent (upper panel in Figure 4c) - a similar pattern to that seen for human genes of similar size (1 to 2 kb). Intragenic DNA methylation in plant genomes is also concentrated in the central region of protein coding sequences (lower panel in Figure 4c). *Arabidopsis *genes share a similar pattern with human genes of similar size (1 to 3 kb). Rice genes are longer than *Arabidopsis *genes (Figure 4d) and have detectable, if not complete, peak patterns, which can explain the observed 5'-end peak [4].

## Discussion

Evolutionary processes seem to have maintained the boundaries of protein coding sequences as targets for nucleosome binding or DNA methylation. Incorporating novel protein domains or introns in inner sequences might have concentrated the epigenetic marks in the 5'- and 3'-end segments. It could be a good strategy to diversify DNA sequences in the middle of coding regions to encode various protein functions while constraining the boundary sequences, including the start and stop codons, to create an epigenetically favorable environment.

What is the physiological role of the conserved 5'- and 3'-end peaks? A few studies have hinted at their role in RNA splicing. First, a crosstalk between nucleosomes and RNA splicing has been suggested [21,22]. The chromatin remodeling complex SWI/SNF was shown to create roadblocks of nucleosomes that slow Pol II elongation, which facilitates the inclusion of the exon during RNA splicing. Another study showed that H3K46me3, which impedes Pol II elongation, was specifically enriched in constitutive exons compared to alternative exons, thus suggesting it has a role in controlling RNA splicing [23,24].

These findings suggest a novel role for nucleosome deposition and DNA methylation at coding boundaries in controlling RNA splicing. The first and last coding exons should be constitutively included in a mature transcript since skipping these exons causes translation failure. Slowing elongation by these epigenetic roadblocks might facilitate the inclusion of these indispensable boundary exons. DNA sequences surrounding the start and stop codons are highly conserved to ensure a favorable epigenetic environment, explaining why the epigenetic peaks are found in the specific loci of the first and last exons. This mechanism seems to be more common among higher organisms with longer genes.

## Conclusions

Previous epigenetic studies have focused on promoter regions in an attempt to associate epigenetic patterns with transcription initiation activity. However, recent genome-wide epigenetic studies challenge the traditional viewpoint and have shed new light on the epigenetic control of transcription elongation. Notably, epigenetic inhibition of Pol II elongation has been proposed to facilitate the inclusion of constitutive exons during RNA splicing. The evolutionarily conserved epigenetic patterns we identified here seem to ensure the inclusion of the first and last coding exons as they are indispensable for accurate translation. Further mechanistic studies are required to support this hypothesis.

## Materials and methods

### Identifying gene structure

The chromosomal positions of transcription initiation and termination sites, and coding start and end sites were obtained from the RefSeq Genes track of the UCSC Genome Browser [25] for the human, mouse, and fly genomes. Each entry was treated as an individual gene. The start and end positions of yeast ORFs were obtained from the *Saccharomyces *Genome Database [26]. The positions of TSSs relative to the start codons of yeast ORFs were obtained from a genome-wide full-length cDNA analysis [27].

### Nucleosomal data for yeast, fly and human

H2A.Z-containing nucleosomes were mapped to the yeast genome [9] and the fly genome [10]. Predictions of nucleosomal locations were downloaded from the authors' website for yeast [28] and fly [29]. The average occupancy of predicted nucleosomes was given as a function of distance from the TSS, start codon, or stop codon across multiple aligned genes. H2A.Z-containing nucleosomes in human T cells were mapped to the human genome by means of Solexa sequencing technology [14]. The tag coordinate files in the browser extensible data (BED) format for resting nucleosomes were downloaded from the supplementary website [30]. The sequencing reads were extended to 150 bp in length according to their orientation [14] and the number of overlapping sequence reads was obtained at 150-bp intervals across the human genome (UCSC hg18 assembly based on NCBI build 36.1). The read counts served as estimates of nucleosome level at the corresponding genomic intervals.

### Affinity purification of methylated genomic DNA

We purified genomic DNA with the aid of the DNeasyR Blood and Tissue Kit (Qiagen, Valencia, CA, USA). The methylated CpG island recovery assay (MIRA) [31] was carried out as follows. Preparation of the glutathione S-transferase (GST)-tagged MBD2b and His-tagged MBD3L1 proteins was done as described [32]. The purified genomic DNA was sonicated to 100 to 500 bp in size and incubated with the prepared GST-MBD2b protein, His-MBD3L1 protein, and the JM110 bacterial DNA. MagneGST beads (Promega, Madison, WI, USA) were pre-blocked with the JM110 bacterial DNA and incubated at 4°C in the MIRA binding buffer (10 mM Tris-HCl, pH 7.5, 50 mM NaCl, 1 mM EDTA, 1 mM dithiothreitol, 3 mM MgCl_2_, 0.1% Triton X-100, 5% glycerol, 25 μg/ml bovine serum albumin) and then washed in washing buffer (10 mM Tris-HCl, pH 7.5, 300 mM NaCl, 1 mM EDTA, 3 mM MgCl_2_, 0.1% Triton X-100). DNA elution was done by incubation at room temperature for 5 minutes and 56°C for 30 minutes with RNase A and Proteinase K. Additional purification of the eluted DNA fragments was done with the aid of the QIAquick PCR Purification Kit (Qiagen).

### Solexa sequencing of affinity-purified methylated DNA

The eluted DNA fragments were ligated to a pair of Solexa adaptors for Illumina Genome Analyzer sequencing. The ligation products were size-fractionated to obtain 175 to 225-bp fragments on an agarose gel and subjected to PCR amplification. Cluster generation and 36 cycles of sequencing were done according to the manufacturer's instructions. The sequence tags were mapped to the human genome (UCSC hg18 assembly based on NCBI build 36.1) or the mouse genome (UCSC mm9 assembly based on NCBI build 37) by means of the Solexa Analysis Pipeline (version 0.3.0). We obtained 34-bp sequenced reads excluding the first and last nucleotide. The raw sequence tags have been deposited in NCBI's Short Read Archive (SRA) under accession number GSE17554.

### Profiling DNA methylation in human T cells and mouse liver

Affinity purification and Solexa sequencing of methylated DNA fragments were carried out for human naïve T cells purified from blood samples from healthy males and females and for liver tissue from normal mice (C57BL/6). To identify the actual positions of methylated DNA fragments, we extended the 34-bp reads toward the 3' end according to the size fractionation of the ligation products (200 bp on average). The output was converted to BED files for visualization in the UCSC genome browser. We counted overlapping sequence tags at a 150-bp resolution for consistency with the nucleosomal data. The read counts served as estimates of methylation level at their respective genomic intervals.

### Obtaining Pol II density data and gene expression data for human T cells, and estimating elongation efficiency

The tag coordinate BED files for unphosphorylated Pol II and Ser5-phosphorylated Pol II in resting T cells were downloaded from [30]. We counted overlapping sequence tags at a 150-bp resolution for consistency with the other data. The read counts served as estimates of Pol II density at their respective genomic intervals. Microarray data for gene expression level in resting T cells are available at the Gene Expression Omnibus (GEO) under accession number GSE10437 [14]. Efficiency of transcriptional elongation was estimated as mRNA production per unit density of elongating Pol II. The expression level of a gene was divided by the average density of Ser5-phosphorylated Pol II over its transcribed region (from the TSS to the termination site). An arbitrary value (0.01) was added to the denominator to avoid illegal division by zero. The ratio was log transformed (base *e*).

### Genetic and epigenetic codes near the start codon and stop codon

For quantitative analysis of genetic or epigenetic codes near coding start and end sites, we used the DNA sequences flanking the coding ends or the 150-bp genomic intervals closest to the coding ends. Nucleosome level, methylation level, and CpG density were obtained from the two to four genomic intervals closest to the start codon or stop codon. CpG density within a genomic interval was obtained as described below. Bending propensity was calculated for the flanking sequences as described below.

### Calculation of DNA bendability and CpG density

DNA-bending propensity was calculated based on trinucleotide parameters [20]. A bending parameter was assigned for each single-nucleotide position according to base composition and then averaged in a sliding window of 100 bp or within a 150-bp segment downstream of the start codon or upstream of the stop codon. The percentage of G and C nucleotides was obtained for the 150-bp intervals. The number of G and C nucleotides was divided by the total number of nucleotides in the segment (that is, 150 nucleotides). CpG density was calculated for the same 150-bp intervals that were used for nucleosomal level estimation and methylation level estimation by the ratio of observed to expected CpG frequencies according to the formula cited in Gardiner-Garden *et al*. [33]. The genomic coordinates of CpG islands were downloaded from the CpG islands track at the UCSC genome browser, as predicted by the following criteria: GC content of 50% or greater, length greater than 200 bp, and a ratio greater than 0.6 of observed number of CpG dinucleotides to the expected number. A gene was deemed to contain a CpG island if the region -1,000 bp to 500 bp from the TSS contained one of the defined CpG islands.

### Methylation data for *Arabidopsis thaliana *and *Oryza sativa*

DNA methylation in the *Arabidopsis *genome was mapped for wild-type roots using tiling microarrays [34]. The data are available at the GEO under accession number GSE12212 (WT root-1 and WT root-2). DNA methylation of rice chromosomes 4 and 10 was mapped using tiling microarrays for cultured cells and light-grown shoots [4]. The data are available at the GEO under accession number GSE9925 (CC replicates 1 to 2 and LS replicates 1 to 2).

### Statistical tests

The length of the 5' UTRs of selected genes was compared against all 5' UTRs in the genome by means of the Wilcoxon rank sum test. Gene expression level was tested by means of the two-sample *t*-test of log ratios or the Wilcoxon rank sum test. Genes with high or low nucleosome occupancy and high or low methylation level at coding boundaries are defined as the top or bottom 10% in each category.

## Abbreviations

GEO: Gene Expression Omnibus; GST: glutathione S-transferase; MIRA: methylated CpG island recovery assay; ORF: open reading frame; Pol II: RNA polymerase II; TSS: transcription start site; UTR: untranslated region.

## Competing interests

The authors declare that they have no competing interests.

## Authors' contributions

JKC conceived of the study, carried out the analysis, and wrote the manuscript. JBB carried out the purification and sequencing of methylated DNA. JL processed the raw sequencing data and participated in data analysis. TYK participated in sample preparation. YJK participated in study design and coordination, and finalized the manuscript.

## Additional data files

The following additional data are available with the online version of this paper: a figure showing nucleosome patterns surrounding the TSS, start codon, and stop codon in yeast and fly (Additional data file 1); a figure showing illustrative genes with nucleosomal peaks at coding boundaries (Additional data file 2); a figure showing DNA methylation level surrounding the transcript and coding region boundaries in the mouse liver (Additional data file 3); a figure showing nucleosome occupancy according to differential Pol II elongation efficiency (Additional data file 4); a figure comparing densities of Ser5-phosphorylated and unphosphorylated Pol II (Additional data file 5); a figure showing Pol II density with higher and lower nucleosome occupancy (Additional data file 6); a figure showing DNA bending propensity at the start and stop codons (Additional data file 7); a figure demonstrating the length of the 5' UTR and gene expression level for genes with high CpG density around the start codon (Additional data file 8); a figure showing the overall patterns of nucleosome occupancy and DNA methylation level inside the protein coding region (Additional data file 9).

## Supplementary Material

Additional data file 1 Nucleosome patterns surrounding the TSS, start codon, and stop codon in yeast and fly. Click here for file

Additional data file 2 Illustrative genes with nucleosomal peaks at coding boundaries. Click here for file

Additional data file 3 DNA methylation level surrounding the transcript and coding region boundaries in the mouse liver. Click here for file

Additional data file 4 Nucleosome occupancy according to differential Pol II elongation efficiency. Click here for file

Additional data file 5 Densities of Ser5-phosphorylated and unphosphorylated Pol II. Click here for file

Additional data file 6 Pol II density with higher and lower nucleosome occupancy. Click here for file

Additional data file 7 DNA bending propensity at the start and stop codons. Click here for file

Additional data file 8 Length of the 5' UTR and gene expression level for genes with high CpG density around the start codon. Click here for file

Additional data file 9 Overall patterns of nucleosome occupancy and DNA methylation level inside the protein coding region. Click here for file
