# Supplementary material for: Nucleosome deposition and DNA methylation at coding region boundaries
Source: Genome Biol. 2009 Sep 1;10(9):R89. doi: 10.1186/gb-2009-10-9-r89 (PMC2768978; doi:10.1186/gb-2009-10-9-r89)
Supplement: Additional data file 1 — Nucleosome patterns surrounding the TSS, start codon, and stop codon in yeast and fly. [file gb-2009-10-9-r89-S1.pdf]

Figure S1

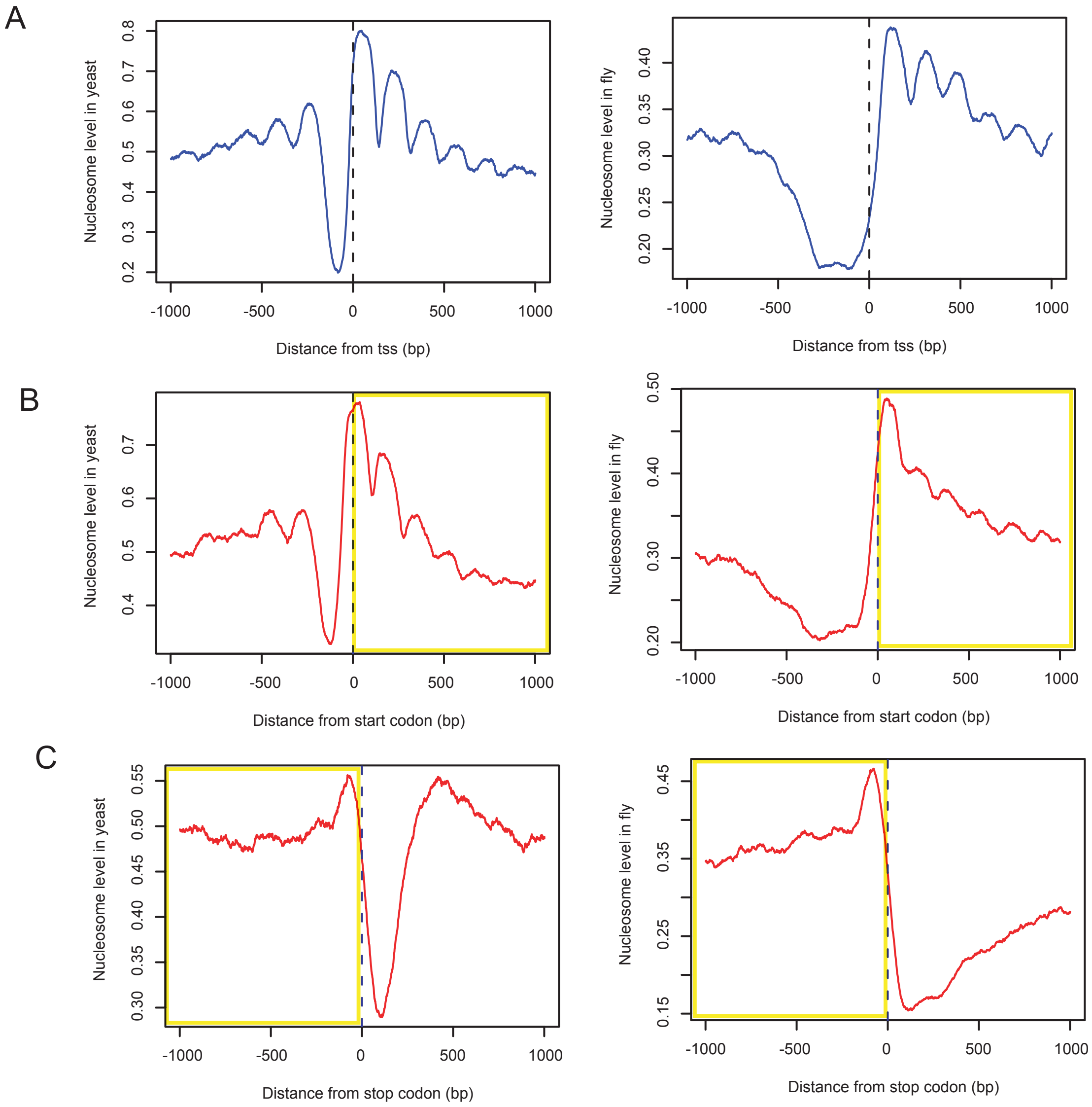

(A) Nucleosome occupancy surrounding the transcription start site. Genomewide average of nucleosome occupancy for yeast (left panel) and fly (right panel) was obtained for genes aligned at the transcription start site.

(B) Nucleosome occupancy surrounding the coding start site of yeast (left panel) and fly (right panel) genes. The inside of the coding region is outlined in yellow.

(C) Nucleosome occupancy surrounding the coding end site of yeast (left panel) and fly (right panel) genes. The inside of the coding region is outlined in yellow.
