# Supplementary material for: Nucleosome deposition and DNA methylation at coding region boundaries
Source: Genome Biol. 2009 Sep 1;10(9):R89. doi: 10.1186/gb-2009-10-9-r89 (PMC2768978; doi:10.1186/gb-2009-10-9-r89)
Supplement: Additional data file 2 — Illustrative genes with nucleosomal peaks at coding boundaries. [file gb-2009-10-9-r89-S2.pdf]

Figure S2

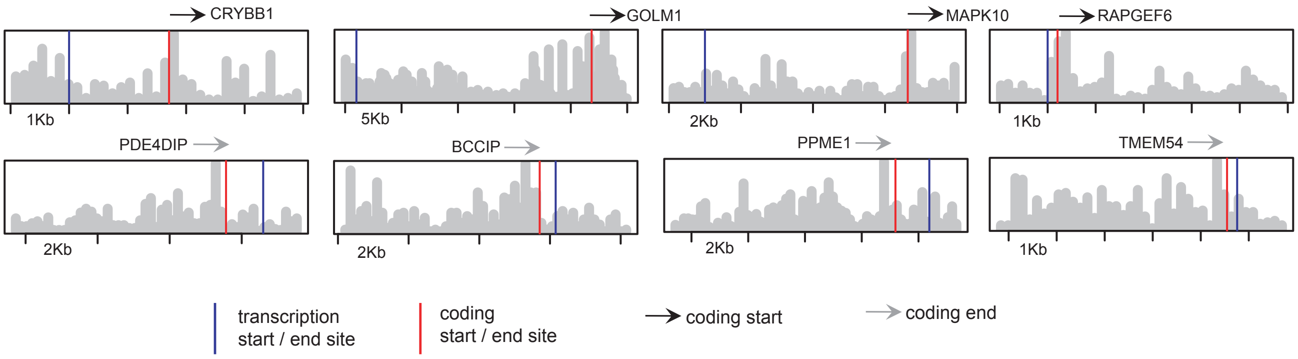

Nucleosome occupancy for genes with a peak downstream from the start codon (CRYBB1, GOLM1, MAPK10, and RAPGEF6) or upstream from the stop codon (PDE4DIP, BCCIP, PPME1, and TMEM54). Coding direction is denoted as an arrow.
