# Supplementary material for: Nucleosome deposition and DNA methylation at coding region boundaries
Source: Genome Biol. 2009 Sep 1;10(9):R89. doi: 10.1186/gb-2009-10-9-r89 (PMC2768978; doi:10.1186/gb-2009-10-9-r89)
Supplement: Additional data file 3 — DNA methylation level surrounding the transcript and coding region boundaries in the mouse liver. [file gb-2009-10-9-r89-S3.pdf]

Figure S3

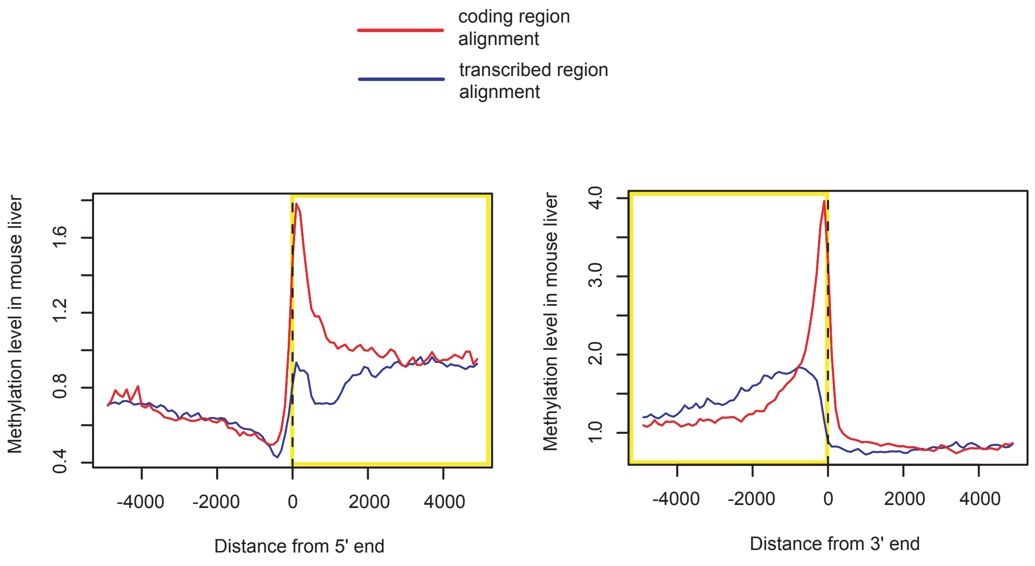

DNA methylation level surrounding the transcript boundaries and coding region boundaries in the mouse liver. Genomewide average of methylation level was obtained for genes aligned at the 5' end (left panel) or at the 3' end (right panel) of the transcript (blue trace) or the coding region (red trace). The inside of the coding region or the transcript is outlined in yellow.
