# Supplementary material for: Nucleosome deposition and DNA methylation at coding region boundaries
Source: Genome Biol. 2009 Sep 1;10(9):R89. doi: 10.1186/gb-2009-10-9-r89 (PMC2768978; doi:10.1186/gb-2009-10-9-r89)
Supplement: Additional data file 4 — Nucleosome occupancy according to differential Pol II elongation efficiency. [file gb-2009-10-9-r89-S4.pdf]

Figure S4

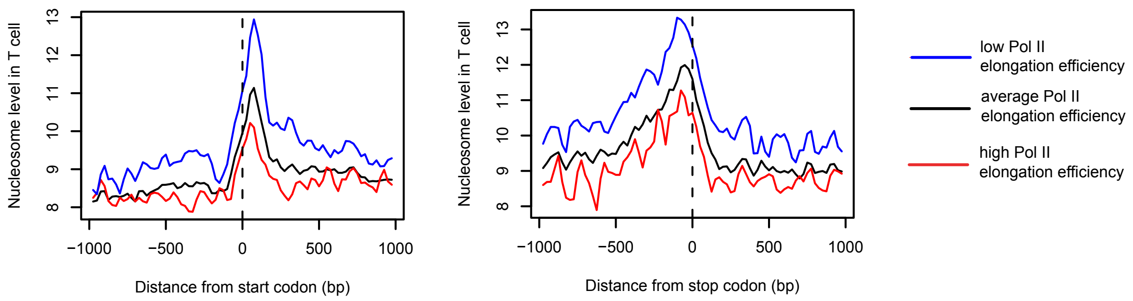

Nucleosome occupancy surrounding the start codon (left panel) and stop codon (right panel) according to differential Pol II elongation efficiency around the start codon and stop codon, respectively. Pol II elongation efficiency was calculated as the ratio of expression level over Pol II density in these regions. Top 20 % and lowest 20 % of elongation efficiency were used as criteria for high and low elongation efficiency.
