# Supplementary material for: Nucleosome deposition and DNA methylation at coding region boundaries
Source: Genome Biol. 2009 Sep 1;10(9):R89. doi: 10.1186/gb-2009-10-9-r89 (PMC2768978; doi:10.1186/gb-2009-10-9-r89)
Supplement: Additional data file 5 — Densities of Ser5-phosphorylated and unphosphorylated Pol II. [file gb-2009-10-9-r89-S5.pdf]

Figure S5

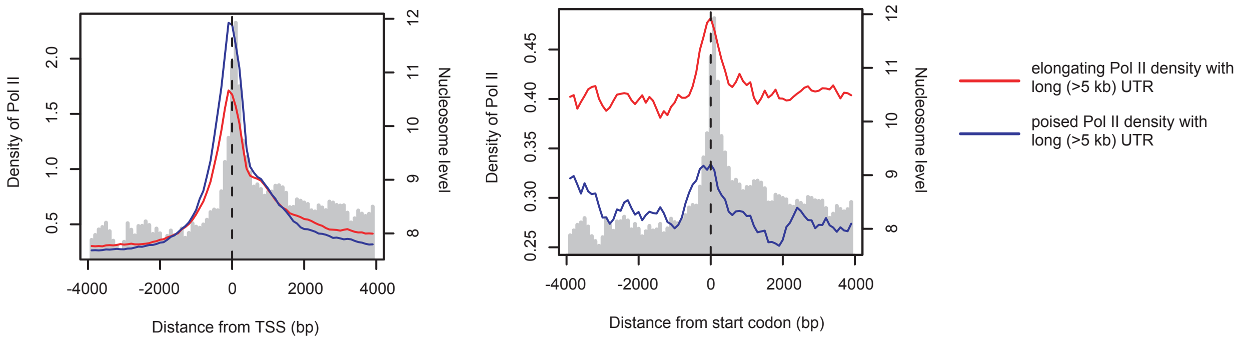

Density of Ser5-phosphorylated Pol II (red trace), density of unphosphorylated Pol II (blue trace), and nucleosome level (gray shade) surrounding the transcription start site (left panel) and start codon (right panel) where the two sites are > 5 kb apart. The Pol II scale is on the left side and the nucleosome scale is on the right side.
