# Supplementary material for: Nucleosome deposition and DNA methylation at coding region boundaries
Source: Genome Biol. 2009 Sep 1;10(9):R89. doi: 10.1186/gb-2009-10-9-r89 (PMC2768978; doi:10.1186/gb-2009-10-9-r89)
Supplement: Additional data file 6 — Pol II density with higher and lower nucleosome occupancy. [file gb-2009-10-9-r89-S6.pdf]

Figure S6

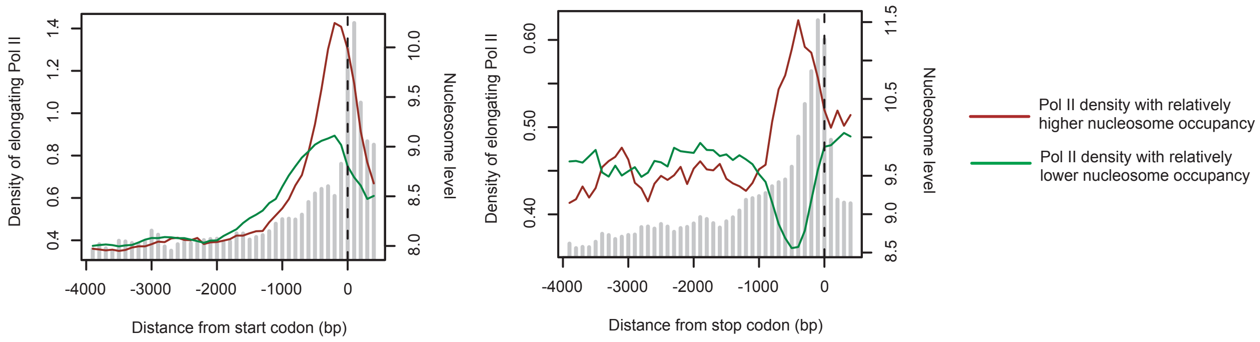

Density of Ser5-phosphorylated Pol II with relatively high- vs. low-occupancy nucleosomes (top 10% vs. lowest 10%) (red trace vs. green trace) and nucleosome level (gray bars) near the start codon (left panel) and near the stop codon (right panel). The Pol II scale is on the left side and the nucleosome scale is on the right side. Relatively high (or low) occupancy means that the occupancy of the boundary nucleosome is higher (or lower) compared to the other nucleosomes in the surrounding region, regardless of absolute occupancy in gene-wise comparison.
