# Supplementary material for: Nucleosome deposition and DNA methylation at coding region boundaries
Source: Genome Biol. 2009 Sep 1;10(9):R89. doi: 10.1186/gb-2009-10-9-r89 (PMC2768978; doi:10.1186/gb-2009-10-9-r89)
Supplement: Additional data file 7 — DNA bending propensity at the start and stop codons. [file gb-2009-10-9-r89-S7.pdf]

Figure S7

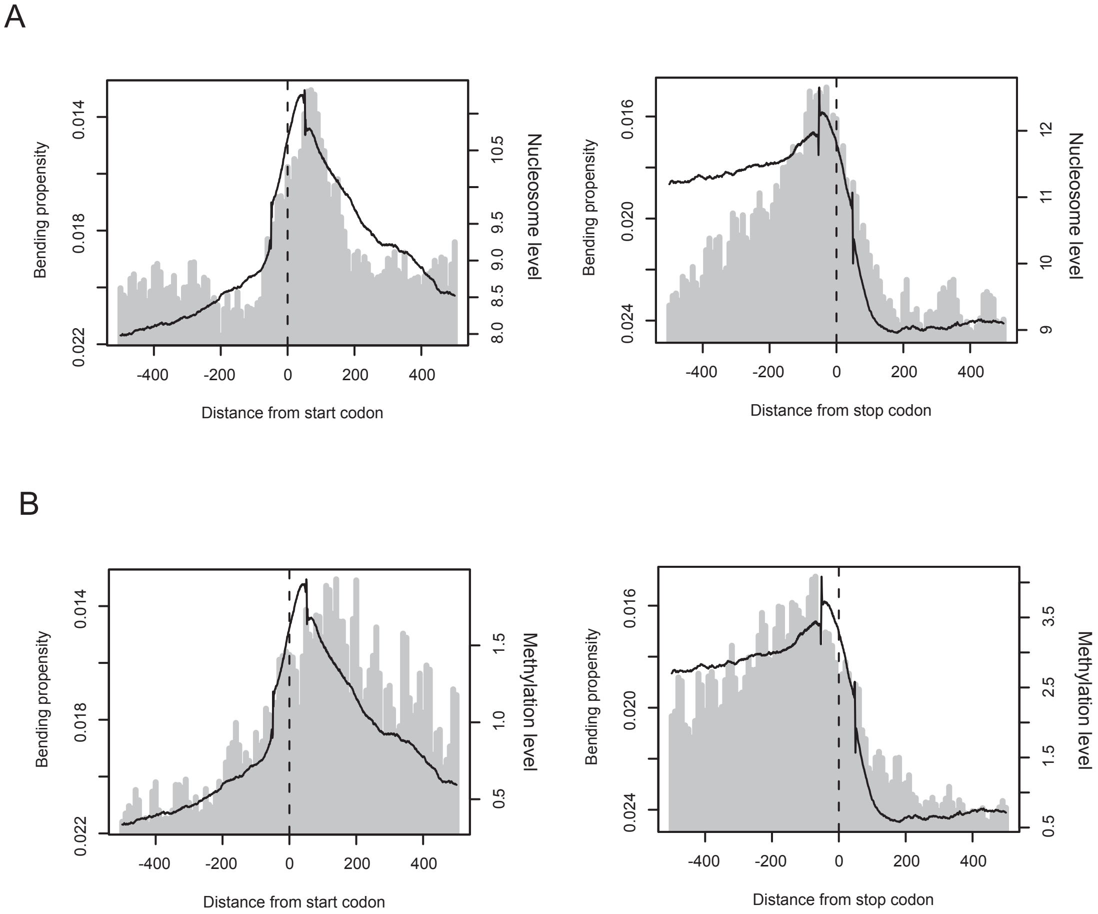

DNA-bending propensity, nucleosome occupancy, and methylation density surrounding the start codon and stop codon. A bending parameter was obtained for each trinucleotide across the surrounding region (see Materials and Methods). For each gene, bending propensity was averaged in a sliding window of 100 nucleotides. Genomewide average of bending propensity (black trace scaled on the left-side axis), nucleosome level (gray shade in A scaled on the right-side axis), and methylation level (gray shade in B scaled on the right-side axis) were obtained for genes aligned at the start codon or stop codon. While correlated with bending propensity, nucleosome level and methylation level appear to be independent.
