# Supplementary material for: Nucleosome deposition and DNA methylation at coding region boundaries
Source: Genome Biol. 2009 Sep 1;10(9):R89. doi: 10.1186/gb-2009-10-9-r89 (PMC2768978; doi:10.1186/gb-2009-10-9-r89)
Supplement: Additional data file 8 — Length of the 5' UTR and gene expression level for genes with high CpG density around the start codon. [file gb-2009-10-9-r89-S8.pdf]

Figure S8

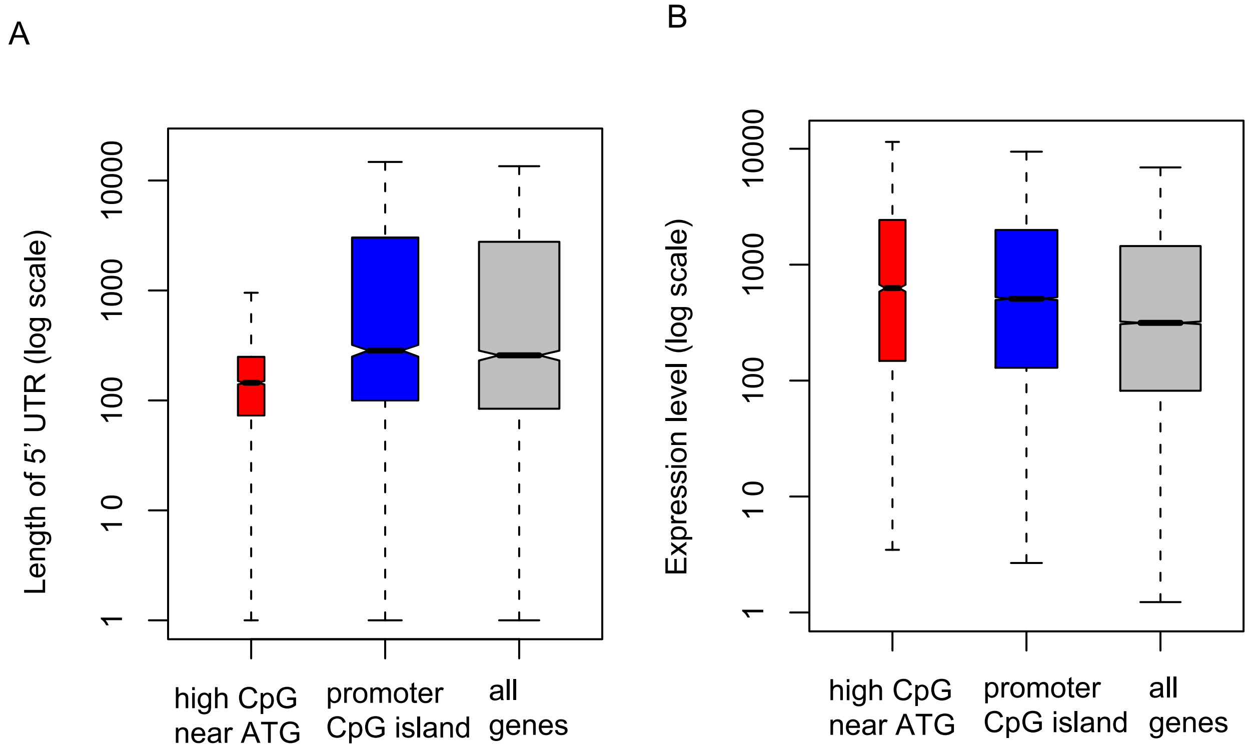

The size of the 5' UTR (A) and gene expression level in human T cells (B) were surveyed for genes with high CpG density near their start codon (2,777 genes) and those with a CpG island in their promoter (17,308 genes), in comparison to all genes in the genome. The y axis is shown on a log scale. The boxes are shown with widths proportional to the square roots of the number of genes in the groups. The whiskers are extended to the most extreme data point which is no more than four times the interquartile range from the box. The outliers are not drawn.
