# Supplementary material for: Nucleosome deposition and DNA methylation at coding region boundaries
Source: Genome Biol. 2009 Sep 1;10(9):R89. doi: 10.1186/gb-2009-10-9-r89 (PMC2768978; doi:10.1186/gb-2009-10-9-r89)
Supplement: Additional data file 9 — Overall patterns of nucleosome occupancy and DNA methylation level inside the protein coding region. [file gb-2009-10-9-r89-S9.pdf]

Figure S9

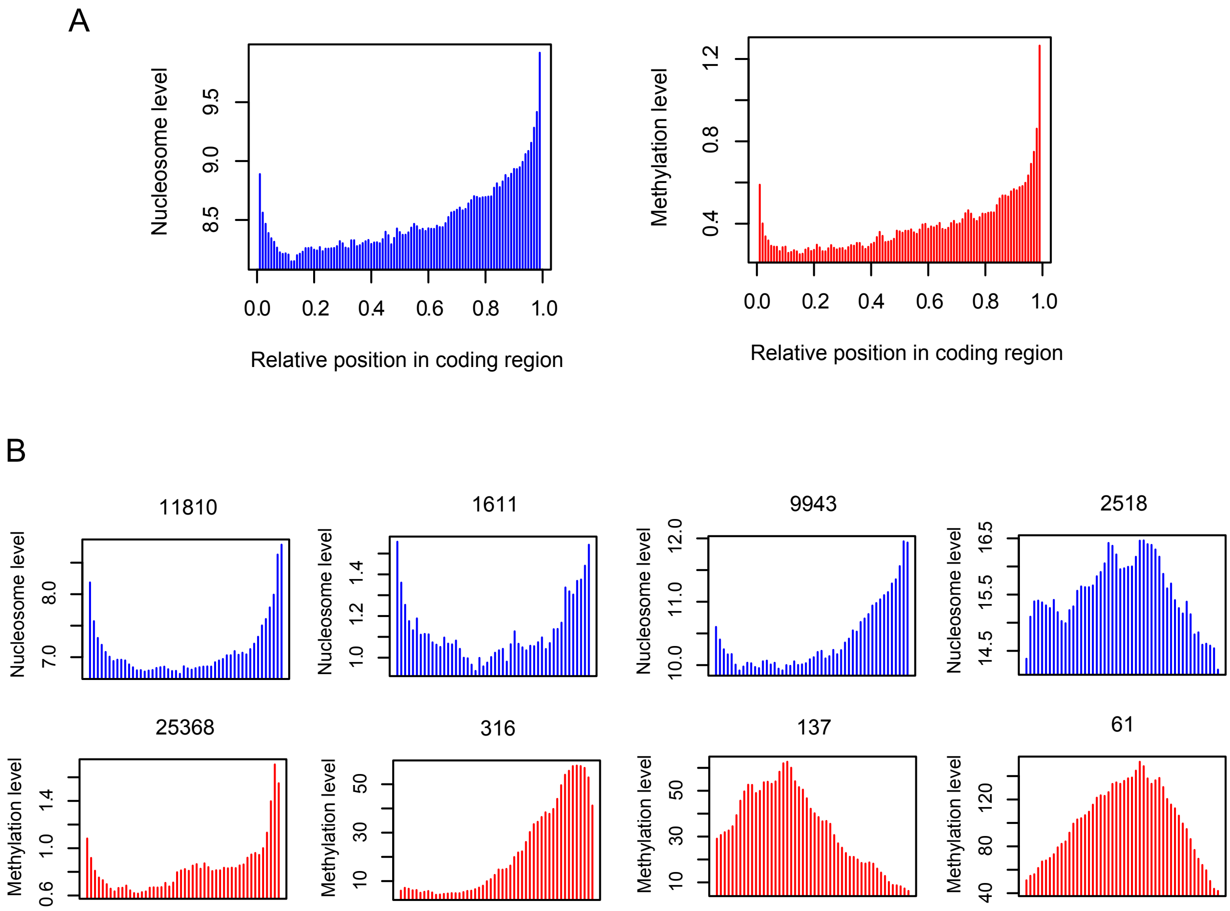

Nucleosome occupancy and DNA methylation level within the coding region.

(A) Genomewide average of nucleosome level (blue bars) and methylation level (red bars) in human T cells was obtained according to relative positions in the coding region.

(B) K-means clustering (k=4) results for the within-coding-region nucleosome profiles (blue bars in upper panel) and for the within-coding-region methylation profiles (red bars in lower panel). The number of genes in each cluster is presented above the plot. The first three nucleosome clusters and the first methylation cluster were considered to have peaks at both ends.
